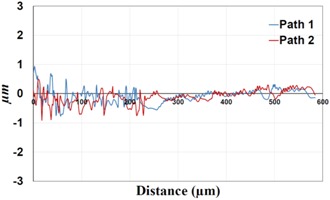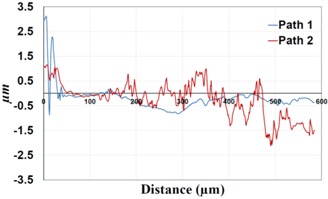# Surface Characterizations of Fretting Fatigue Damage in Aluminum Alloy 7075-T6 Clamped Joints: The Beneficial Role of Ni–P Coatings

**DOI:** 10.3390/ma9030141

**Published:** 2016-02-27

**Authors:** Reza H. Oskouei, Mohammad Reza Barati, Raafat N. Ibrahim

**Affiliations:** 1 Discipline of Mechanical Engineering, School of Computer Science, Engineering and Mathematics, Flinders University, Clovelly Park SA 5042, Australia; mohammad.barati@flinders.edu.au; 2 Department of Mechanical & Aerospace Engineering, Monash University, Clayton VIC 3800, Australia; raafat.ibrahim@monash.edu; 3 Department of Materials Science and Engineering, Monash University, Clayton VIC 3800, Australia

**Keywords:** fretting fatigue, aluminum alloys, Ni–P coatings, surface roughness, clamped joints

## Abstract

This paper aims to characterize the surface damage as a consequence of fretting fatigue in aluminum alloy 7075-T6 plates in double-lap bolted joints through XRD, surface profilometry, and SEM analyses. The main focus was on the surface roughness and chemical phase composition of the damaged zone along with the identification of fretting fatigue crack initiations over the surface of the material. The surface roughness of the fretted zone was found to increase when the joint was clamped with a higher tightening torque and tested under the same cyclic loading. Additionally, MgZn_2_ (η/ή) precipitates and ZnO phase were found to form onto the surface of uncoated aluminum plate in the fretted and worn zones. The formation of the ZnO phase was understood to be a result of frictional heat induced between the surface of contacting uncoated Al 7075-T6 plates during cyclic loading and exposure to the air. The beneficial role of electroless nickel-phosphorous (Ni–P) coatings in minimizing the fretting damage and thus improving the fretting fatigue life of the aluminum plates was also studied. The results showed that the surface roughness decreased by approximately 40% after applying Ni–P coatings to the Al 7075-T6 plates.

## 1. Introduction

Mechanical and/or structural elements, which are clamped together and withstand small-amplitude oscillatory motions, may face an early failure as a result of fretting fatigue [1]. This phenomenon is very important, particularly in aerospace structures. In these situations, local surface adhesions between the contacting materials initiate fretting damage; subsequently, material removal occurs over the surface [2]. Mechanically fastened joints have been found as a common situation where fretting fatigue can occur, resulting in a significant reduction in fatigue life when subjected to cyclic loads [3,4]. Despite the beneficial role of high clamping forces in reducing stress concentration around the fastener hole [5,6,7], which can then help to improve the fatigue life [8,9], fretting fatigue can unfavorably accelerate under high clamping forces [10,11]. Benhamena *et al.* [10] reported significant reductions in the fretting fatigue life of a single-lap bolted joint composed of aluminum alloy and steel plates as a result of increasing the tightening torque. The increase in torque was reported to cause frictional stresses to increase and relative slip to reduce at the interface. Energy dispersive spectroscopy (EDS) analyses showed a reduction in the amount of aluminum element and an increase in the amount of iron and silicon onto the damaged surface, although the mechanism of this chemical composition change was not addressed. Shinde and Hoeppner [12] characterized fretting damaged zones in Al 7075-T6 specimens tested under a contacting pad. Cracks were found to initiate at pits due to the adhesive contact of asperities and oxides. Fretting degradation was reported to depend on the microstructure of the material such that smaller grain sizes had more resistance to fretting, whereas larger grain sizes were more vulnerable.

Mitigation of fretting fatigue is of paramount importance with the aim of improving fatigue performance of contacting materials to avoid catastrophic failures in structures. Surface modification methods, particularly coatings, have been employed to modify the fretting behavior of materials [4,13,14,15,16]; however, a better understanding of the damage mechanism is needed to apply a suitable coating which can minimize the risk of fretting fatigue failures. In a previous study [4], fretting fatigue life was examined in a double-lap bolted joint made of Al 7075-T6 plates. Fatigue life was found to reduce as the tightening torque increased and the cyclic load level decreased. Electroless Ni–P coatings with a thickness of 40 µm beneficially improved the fretting fatigue life by 30%–40% and 50%–60% for the joints clamped with 5 Nm and 8 Nm, respectively. However, the origin and morphology of the fretting fatigue damage on the surface of the aluminum alloy were not characterized. Additionally, the role of coating in prevention of the fretting fatigue damage formation was not fully addressed.

In this work, characteristics of fretting fatigue damage and the extent of damaged zones over Al 7075-T6 plates were studied in a double-lap bolted joint configuration. The mechanism of fatigue crack initiations from the fretting damage was investigated. Surface topography, roughness and chemical phase composition of the material surface were characterized before and after fatigue tests with the aim of understanding the effective mechanism for surface degradation and debris formation. The beneficial effect of Ni–P coatings was also investigated in terms of surface roughness and formation of new chemical compounds onto the surface. The outcomes of this work can help to better design and/or modify mechanically fastened joints in fretting fatigue situations such as aerospace structures.

## 2. Experimental Details

**Figure 1:**
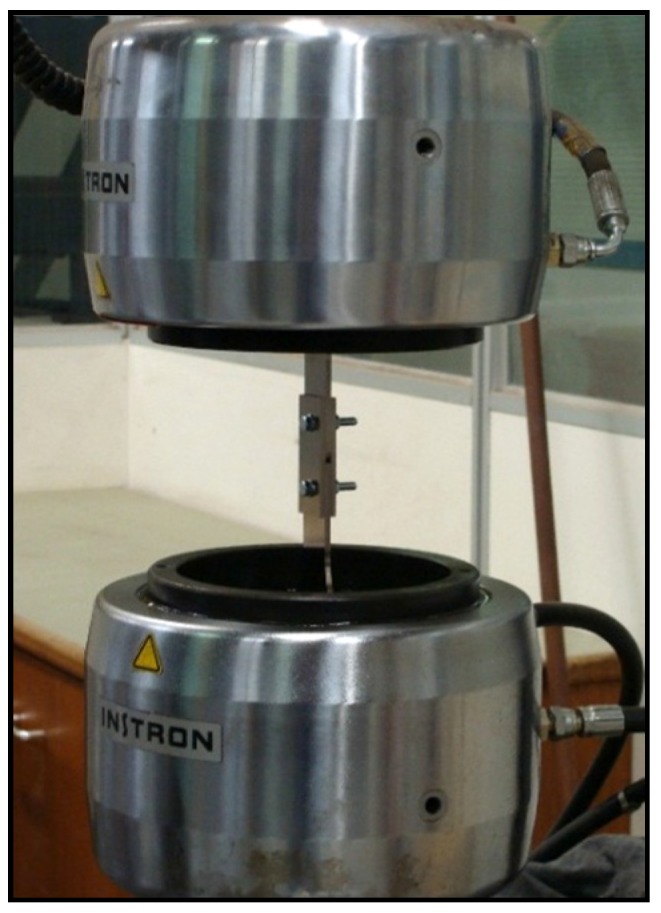
Aluminum alloy 7075-T6 double-lap bolted joint specimen under fatigue loading.

Fatigue and fretting fatigue lives of a double-lap bolted joint specimen composed of aluminum alloy 7075-T6 plates with a thickness of 3.175 mm (two middle plates and two connecting plates), and two M5 steel bolts (Figure 1) were reported in the previous work [4]. Using a calibrated torque wrench (Norbar, Model SL0, Willoughby, OH, USA) with a torque range of 1–20 Nm and ±4% accuracy, two tightening torques of 5 Nm and 8 Nm were applied equally to both bolts in order to clamp the plates. For the joint specimens with coating, the Al 7075-T6 plates were coated with electroless nickel-phosphorous (Ni–P) coatings with a high phosphorous content of 10–13 wt % and a thickness of approximately 40 µm using electroless nickel plating (EN) method at Reliable Plating Corp, Chicago, IL, USA. The chemical composition of the Al 7075-T6 substrate (in wt %) was 5.7 Zn, 2.7 Mg, 1.4 Cu, 0.01 Mn, 0.11 Fe, 0.07 Si, 0.02 Ti, 0.01 Zr and 0.19 Cr (Kaiser Aluminum, Spokane, WA, USA).

**Figure 2:**
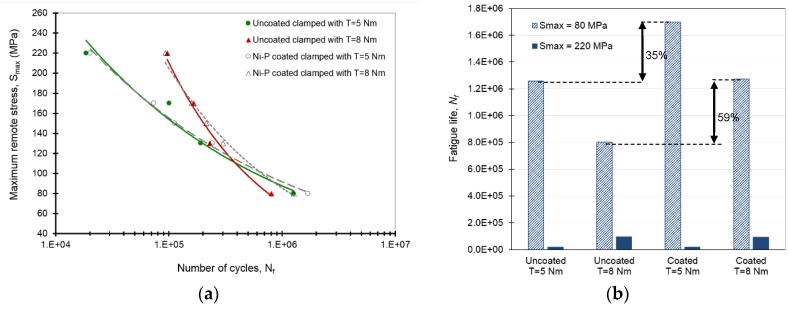
(**a**) Fatigue life results for uncoated and Ni–P coated aluminum alloy 7075-T6 joints clamped with 5 and 8 Nm; and (**b**) a comparison between fatigue life for the joint specimens that failed at two cyclic load levels with maximum remote stresses of 80 and 220 MPa. The joint specimens were tested under constant-amplitude sinusoidal loading at 10 Hz with a load ratio of 0.1 (results extracted from previous work [4]).

Fatigue life results are shown in Figure 2a in the form of S-N curves. For each data point, three fatigue tests were carried out and the average fatigue life was used. The coefficient of determination (*R*^2^) for the S-N curves was above 0.97 with the upper and lower confidence bounds of 95% [4]. The results showed that, at high and moderate cyclic load levels (maximum remote stresses, *S_max_* > 130 MPa), the higher clamping force could increase the fatigue life in both the uncoated and coated joints because of the more compressive stresses around the hole. In these cases, fatigue crack initiations occurred at the critical edge of the hole. However, at low cyclic load levels (*S_max_* < 130 MPa), the uncoated joints faced fretting fatigue failure and thus a reduction in fatigue life. The fatigue life results for two cyclic load levels with maximum remote stresses of *S_max_* = 80 MPa and 220 MPa are given in Figure 2b. Under *S_max_* = 220 MPa, the higher tightening torque (8 Nm) increased the fatigue life, although coatings did not considerably influence the life. However, the higher torque caused less number of cycles to failure under *S_max_* = 80 MPa. Ni–P coatings were found to improve the fretting fatigue life of 5 Nm and 8 Nm clamped joints at low cyclic loads, particularly under *S_max_* = 80 MPa, by 35% and 59% respectively [4].

The contacting region of the middle plates from the eight joint specimens (uncoated and coated aluminum plates clamped with 5 Nm and 8 Nm and tested under *S_max_* = 80 MPa and 220 MPa) were cut and prepared for damage characterization investigations. X-ray diffraction (XRD) analyses were performed for phase identifications of the fretted and worn surfaces using a Philips PW 1140 diffractometer with nickel-filtered Cu Kα radiation (λ = 1.5406 Å). The step size and scanning rate were adjusted as 0.01°/min and 0.1°/min, respectively. 3D surface topography of the damage induced by the interaction between the contacting plates during cyclic loading was investigated by a Veeco Wyko NT1100 optical profilometer (Plainview, NY, USA). The arithmetic height (*R_a_*) of 2D surface roughness profiles in longitudinal direction was measured around the hole of the plates for uncoated and coated specimens. Measurements were performed at five different areas over the damaged zone of each specimen. Roughness *R_a_* was then calculated as the average of these five measurements.

To detect fatigue crack initiation sites and characterize the fretting damage, scanning electron microscopy (JEOL JSM-840A SEM, Peabody, MA, USA) was employed at an accelerating voltage of 20 kV. The elemental percent composition of Ni–P coatings was evaluated by an integrated energy dispersive spectroscopy (EDS) analysis with an ultra-thin window for a light element analysis using a JEOL JSM-840A SEM (Peabody, MA, USA) at 15 kV.

## 3. Results and Discussion

Macroscopic investigations of the uncoated and coated samples showed significant wear damage around the hole of the uncoated samples in both tightening torques under the high cyclic load of 220 MPa. The wear was developed parallel to the loading direction and relative displacement between the plates. The wear damage was substantially alleviated in coated samples. Additionally, fretting fatigue damage was macroscopically observed over the surface of the uncoated samples in both tightening torques under *S_max_* = 80 MPa. The damaged points were located away from the critical edge of the hole (90°). However, coated samples did not possess any fretting fatigue damage onto the surface; instead, the failure was from the hole’s edge. Surface damage characterizations are presented and discussed in the following sections. It is also noted that the presence of Ni–P coatings may decrease the frictional forces acting between the washers and connecting plates. It is believed that such decreased frictional forces may have inconsiderable effect on the actual preloads developed by the tightening torques.

### 3.1. XRD Analysis of Uncoated Al 7075-T6

**Figure 3:**
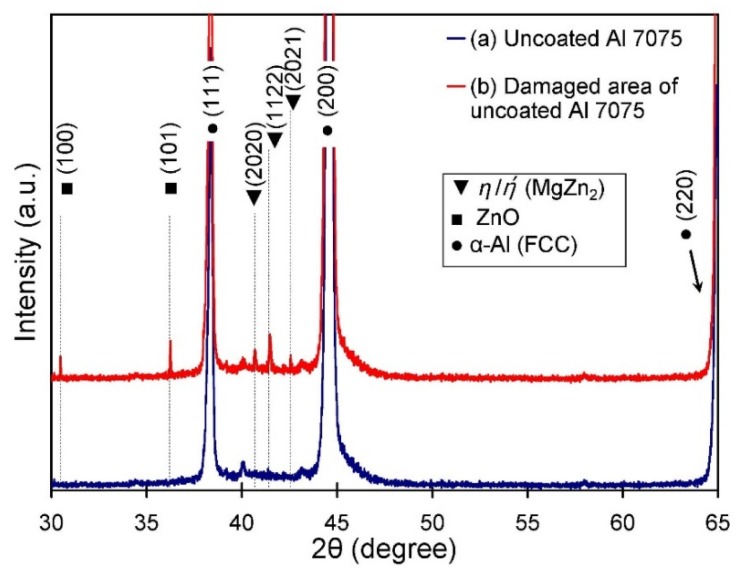
X-ray diffraction patterns of: (**a**) uncoated Al 7075-T6; and (**b**) damaged area of uncoated Al 7075-T6.

To verify the formation of secondary phases on the surface of the uncoated and coated aluminum plates before and after fatigue tests, XRD experiments were conducted. The XRD patterns for the uncoated Al 7075-T6 plates in both the undamaged (before fatigue test) and damaged areas are shown in Figure 3. For the undamaged sample, the strong peaks located at angles 2θ = 38.33°, 44.58° and 64.91° were related to α-Al (FCC), according to JCPDS card #89-4184. The XRD analysis confirmed that α-Al (FCC) phase exists as the dominate phase in the alloy. This indicates that the observed α-Al solid solution phase was free of precipitation. However, the XRD pattern for the damaged area of the uncoated samples that were clamped with 5 Nm and 8 Nm and that failed under *S_max_* = 80 MPa and 220 MPa displayed the presence of some secondary phases in addition to the α-Al (FCC) phase. These secondary phases included weak reflection peaks of MgZn_2_ (η/ή) precipitates and ZnO phase (JCPDS card #75-0576). The X-ray diffraction patterns did not reveal differences in the crystal structure of η and ή precipitates in the uncoated samples due to the overlap between their reflection peaks. Thus, the η and ή phases were evaluated together. The XRD peaks indicated that the η and ή precipitates, which had been initially dissolved in original Al 7075 (during the solutionizing step of T6), started growing under cyclic loading. The formation of ZnO phase could be as a direct result of oxidation reaction. This is an expected phenomenon where the temperature is locally increased by frictional heat induced between the contacting plates during loading and exposure to the air [17,18]. The results demonstrated that these phase composition changes subsequently led to an increase in the number of such hard and brittle compounds on the top surface, which can provide perfect sites for fretting wear and crack initiations [19].

### 3.2. XRD Analysis of Coated Al 7075-T6

**Figure 4:**
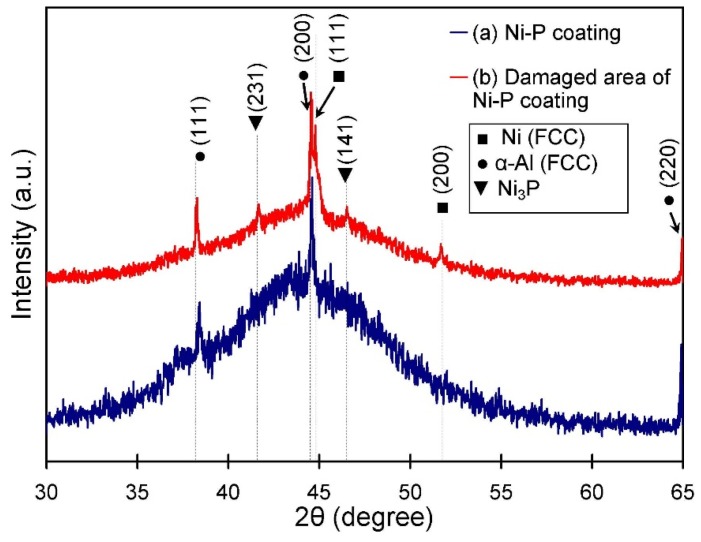
X-ray diffraction patterns of: (**a**) electroless Ni–P coatings; and (**b**) damaged area of Ni–P coatings.

The phase compositions of the coated Al 7075-T6 before and after fatigue tests are presented in Figure 4. The XRD patterns of as-deposited coatings on the substrate before fatigue tests revealed a single broad peak at 2θ ≈ 45°. This indicates Ni (FCC) phase contributions from Ni–P coatings. This was confirmed, as the presence of the broad peak corresponding to the diffraction along (111) was in accordance with cubic structure of Ni (FCC) phase, which was in agreement with JCPDS card #70-0989 with no extra phase. It should be also noted that, although the XRD pattern showed only a main characteristic peak for Ni (FCC), other diffractions along (200) and (220) identical to the standard diffraction pattern data of pure Ni were absent. This suggests a disordered and non-crystalline structure for as-deposited Ni-P coatings on the Al 7075-T6 substrate. It has been confirmed by Kang *et al.* [20] that, in Ni–P coatings, phosphorous is present in the crystal structure of Ni through solid solution, and a higher amount of phosphorous can depress the crystallization of the Ni–P deposition. The EDS analysis on the coating in this study confirmed the presence of phosphorous in a high amount of 11.9 wt %, confirming a non-crystalline structure for the Ni–P coating. It is also noted that the sharp reflection peaks detected at 2θ = 38.33°, 44.58° and 64.91° were related to the aluminum substrate under the coating layer.

As shown in Figure 4, there were some crystalline phases crystallized from the amorphous coating after fatigue tests. Some of those reflection peaks were matched with the standard X-ray diffraction pattern data of Ni_3_P crystal structure (JCPDS card #89-2743). In addition, a small contribution from the reflection peaks with low intensity at 2θ = 44.6° and 51.9° was related to crystallized Ni (FCC) phase in the coating material. The phase composition of amorphous Ni*_x_*P*_y_* has been previously characterized by other researchers [21,22]. Previous studies have shown that amorphous Ni–P may crystallize to Ni_3_P phase upon heating without any additional phosphorous added [23]. This suggests that the frictional heat, induced during cyclic loading, provided sufficient temperature for phase transformation (Ni → Ni_3_P). Figure 4 also shows that crystallization of Ni (FCC) phase in the amorphous Ni–P deposition was improved after the fatigue test. This indicates that, when Ni_3_P precipitates, the amount of phosphorous is reduced in the amorphous Ni–P coating, and improves the crystallization of Ni (FCC) phase. The crystallized Ni phase can be observed in the XRD pattern shown in Figure 4 where the crystallographic planes of (111) and (200) belonging to the Ni (FCC) phase are intensified after fatigue test. This therefore resulted in the formation of a mixture of Ni (FCC) + Ni_3_P in the microstructure of the coating material.

It is noted that the fatigue tests were conducted at a constant frequency of 10 Hz, as reported in the previous study [4]. The amount of frictional heat generation is frequency dependent, and the testing frequency can change the rate of heat generation with time [24,25]. To understand how considerably the frequency can affect the chemical phase composition of the fretting fatigue damaged zones, further investigations are recommended.

### 3.3. Surface Profilometry

Mechanical damage onto the surface of the uncoated specimens was in conjunction with the frictional energy dissipation in the contact areas which was accompanied by temperature increase. It is widely accepted that isomorphous phase MgZn_2_ (η/ή) exists as a strengthening precipitate in Al 7075-T6. However, frictional heat of the contacting plates during cyclic loading resulted in the formation of overaged zones; consequently, growth in the strengthening precipitates, as detected in the form of a secondary phase within the α-Al (FCC) matrix (Figure 3). The growth of MgZn_2_ (η/ή) precipitates from the solute rich solid solution reduces the mechanical properties of the matrix [26]. Therefore, the hard and coarse precipitates are surrounded by a soft and low strength matrix on the surface, which can be easily damaged by hard debris and detached particles from the surface. As another consequence of frictional heat, uncoated bolted joints that had experienced cyclic loads generated a brittle oxide phase (ZnO) on the surface, as detected by the XRD analysis. This brittle oxide phase can have distractive effects when they are removed and comminuted under cyclic loads. The oxide phase on the top surface was subjected to repeated compaction and fragmentation; as a result, oxidative debris was detached from the surface.

**Figure 5:**
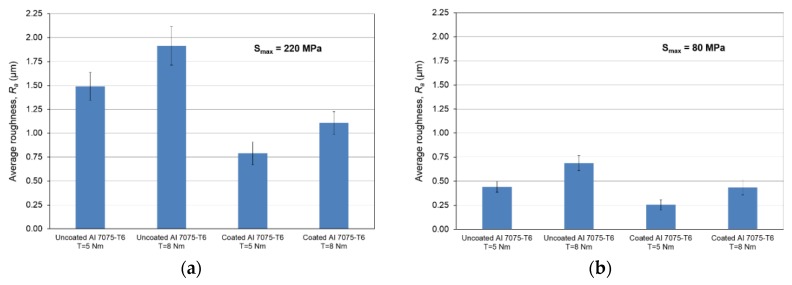
Average roughness *R_a_* for uncoated and coated aluminum plates clamped with 5 Nm and 8 Nm that failed under: (**a**) *S_max_* = 220 MPa; and (**b**) *S_max_* = 80 MPa.

Under *S_max_* = 220 MPa, where the specimens failed from the hole edge (due to the stress concentration), the plate surface well around the hole was influenced by wear (gross sliding). The surface damage in the form of wear can be seen in Table 1. In these specimens, the presence of the hole and its associated stress concentration was the dominant mechanism for fatigue crack initiations, and the worn region did have little effect on the failure of the joint. It can be seen that the roughness profile within the damaged zone was strongly influenced by the tightening torque. The average *R_a_* parameter of the damaged area in the vicinity of the hole for uncoated specimens increased by 28% with an increase in tightening torque from 5 to 8 Nm, as shown in Figure 5a. This was because of the increase in the applied normal force from the bolts towards the contacting plates and consequently a greater friction force between the plates, resulting in more frictional heat. It can be seen from Table 1 that there are deep and oriented grooves on the surface. It seems that the possible mechanism for such a worn surface could be the physical displacement of the contacting material and the ploughing action of the hard compounds such as oxidative debris. It is noteworthy that the high maximum remote stress can intensify the frictional heating effect, softening the matrix. In these cases, deep grooves were formed on the surface of the uncoated plates clamped with 5 Nm, some of which might have coalesced and formed deep pits in the worn area under the higher tightening torque (8 Nm). Ni–P coatings decreased the surface roughness in both the 5 Nm and 8 Nm clamped plates by 47% and 42%, respectively (Figure 5a). This beneficial effect in smoothing the roughness profile within the damaged zone can be also seen in Table 1 for specimens coated with Ni–P.

In bolted joints that failed due to fretting fatigue (those that failed under *S_max_* = 80 MPa), the aluminum plates experienced significant fretting damage over the contacting surface all around the fastener hole. The intensity of wear damage around the hole was much less than the joints that failed under *S_max_* = 220 MPa, as shown in Table 1 and Table 2. In these cases, deep grooves were not formed and material removals occurred in the contact region, resulting in fretting fatigue damage distributed around the hole even at locations away from the hole. Owing to decreased oscillatory displacement amplitudes between the uncoated surfaces, the frictional heating effect, and consequently the resultant debris oxidation, reduced. In addition, separation of oxide phase (ZnO) and scratching of the soft matrix by abrasive oxide particles could be diminished on the surface, as indicated in the surface profiles within the damaged zone (Table 2). Comparing Figure 5a,b, the average roughness (*R_a_*) in both the uncoated and coated plates of 5 Nm and 8 Nm decreased significantly when the maximum remote stress was reduced from 220 MPa to 80 MPa. Additionally, an increase of 56% in the average *R_a_* value can be observed when the uncoated plates were clamped with 8 Nm in comparison with 5 Nm, similar to the roughness trend observed in joints that failed under *S_max_* = 220 MPa (Figure 5a).

As shown in Figure 2b, Ni–P coatings successfully increased the fretting fatigue life of Al 7075-T6 in both the tightening torques under *S_max_* = 80 MPa. The coated joints in these cases failed from the edge of the hole with no fretting fatigue crack initiations on the surface [4]. The surface profilometry showed that the contribution of fretting fatigue damage to the surface roughness was prevented, and roughness was only due to the slight wear damage that occurred around the hole in the coated plates. As can be seen in Figure 5b, the average roughness was reduced by 42% and 37% in 5 Nm and 8 Nm joints, respectively, after coating.

### 3.4. SEM Examinations

As discussed previously, among the eight samples studied in this work, two samples faced fretting fatigue failure. They include uncoated aluminum plates clamped with 5 Nm and 8 Nm that failed under *S_max_* = 80 MPa, where the failure originated from the top surface of the plates at locations away from the hole’s circumference. However, the other samples failed from the critical edge of the hole due to the effect of stress concentration. Given the significance of characterization of fretting fatigue damage, SEM examinations for these two samples were limited to identifying crack initiations from the fretted zones and crack propagations over the fracture surface.

**Figure 6:**
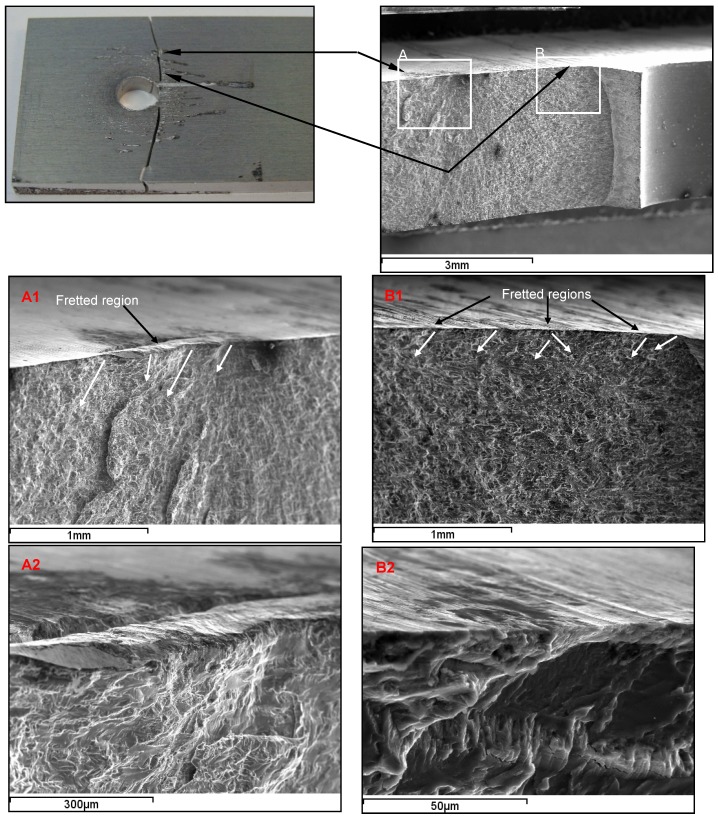
Fretting fatigue damage zones and crack nucleation sites; uncoated Al 7075-T6 plate clamped with *T* = 5 Nm that failed under *S_max_* = 80 MPa. (**A** and **B**) fretted regions; (**A1**, **A2**, **B1** and **B2**) fretted regions at higher magnifications.

**Figure 7:**
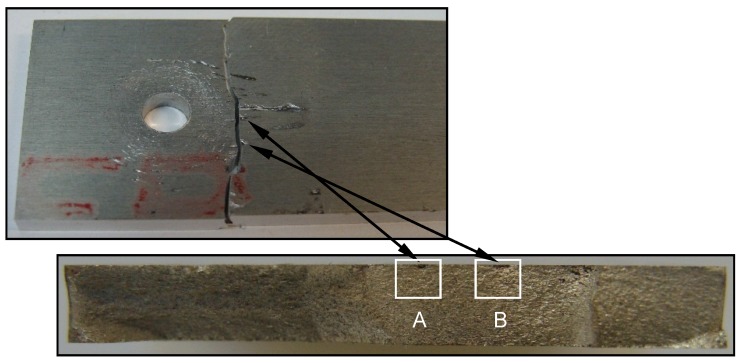

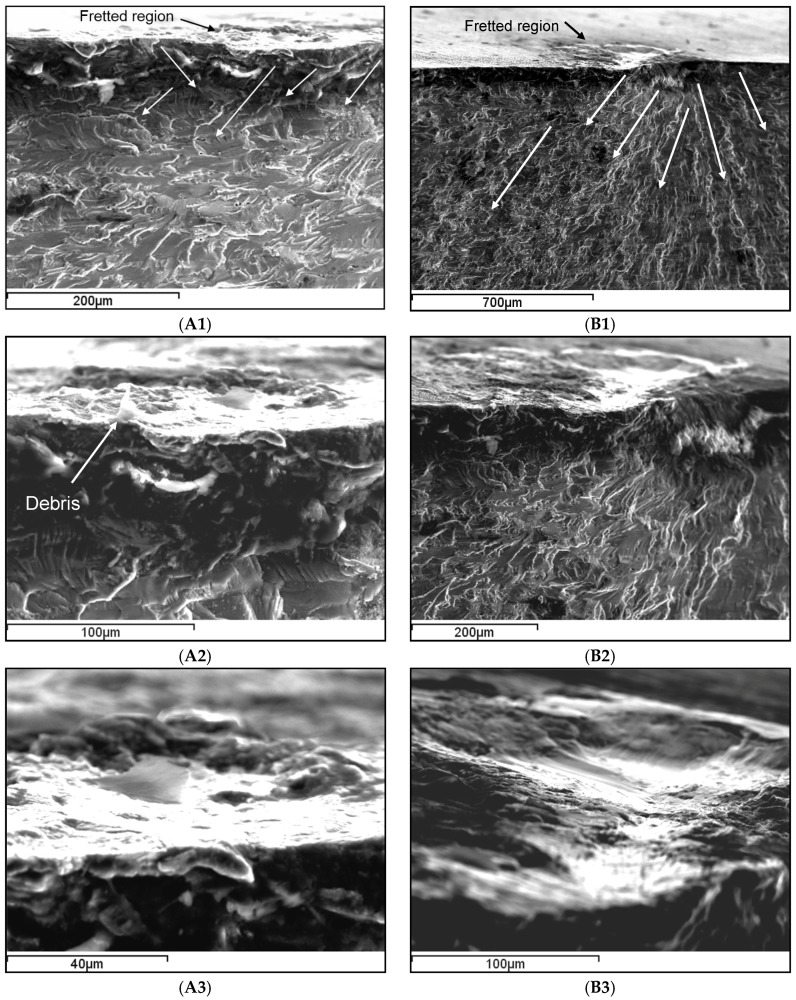
Fretting fatigue damage zones and crack nucleation sites; uncoated Al 7075-T6 plate clamped with *T* = 8 Nm that failed under *S_max_* = 80 MPa. (**A** and **B**) fretted regions; (**A1**–**A3** and **B1**–**B3**) fretted regions at higher magnifications.

In Figure 6 and Figure 7, fretting fatigue damage is displayed for the uncoated plates clamped with 5 Nm and 8 Nm, respectively, that failed under *S_max_* = 80 MPa. The SEM images clearly illustrate the role of fretting damage in initiating fatigue cracks from the top surface. Figure 6 shows multiple-damage sites from which sites A and B are further evaluated. The direction of growth of the fretting cracks is associated with the direction of contact stresses in the fretting area, as indicated by arrows in Figure 6A1,B1. Crack origins in the fretted regions are then shown at higher magnifications in Figure 6A2,B2, where depth and area of material removals can be also seen. Crack initiation sites and crack propagation pathways towards the middle plane of the plate thickness can be observed in these images. In a similar way, cracks initiated from the fretted zones in the case of 8 Nm, as shown in Figure 7. However, fretting damage was found to be more significant in terms of size and depth compared with 5 Nm joints. Unlike the surface scratches present in the 5 Nm plate (Figure 6B2), there were grooves and deeper pits formed in the fretted regions of the plated clamped with 8 Nm (Figure 7B3). These observations are found to be in agreement with the surface profilometry results. The sign of detached debris can be clearly observed in one of the fretted zones, as depicted in Figure 7A2.

## 4. Conclusions

Wear and fretting fatigue damage to Al 7075-T6 plates in a double-lap bolted joint clamped with two tightening torques of 5 Nm and 8 Nm were characterized. The aluminum alloy was influenced by gross sliding and possessed significant wear damage around the hole when subjected to a high cyclic load with a maximum remote stress of 220 MPa. Frictional heat induced between the contacting uncoated Al 7075-T6 plates resulted in the formation of MgZn_2_ (η/ή) precipitates and ZnO phase in addition to the α-Al (FCC) phase. The oxide phase that was separated from the soft matrix was found to be responsible for causing surface scratches as debris and contributing to the roughness of the damaged regions. The average roughness increased with the increase in tightening torque and the maximum remote stress of the cyclic load. Ni–P coatings prevented the formation of fretting fatigue damage onto the surface and were only influenced by slight wear damage observed around the hole. The frictional heat between the coated plates also promoted a phase compositional change on the surface of Ni–P coatings. The appearance of XRD-detectable crystalline phases indicated the crystallization of Ni (FCC) and Ni_3_P phases from the amorphous coating.

## Figures and Tables

**Table 1:** 3D surface profile together with line scan profile for uncoated and coated aluminum plates clamped with 5 Nm and 8 Nm that failed under maximum remote stress of *S_max_* = 220 MPa.

Profiles	Uncoated Al 7075-T6 Plates	
	*T* = 5 Nm	*T* = 8 Nm
3D surface profile	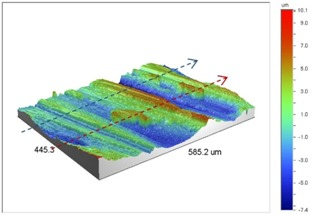	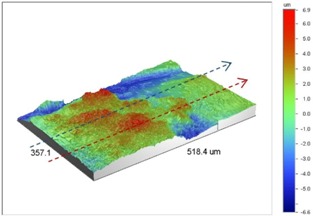
Line scan profiles	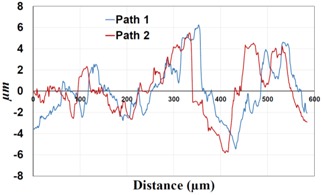	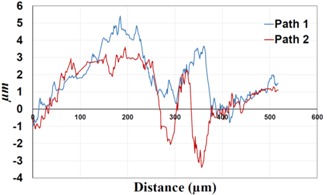
**Profiles**	**Coated Al 7075-T6 plates with Ni–P coatings**	
	***T* = 5 Nm**	***T* = 8 Nm**
3D surface profile	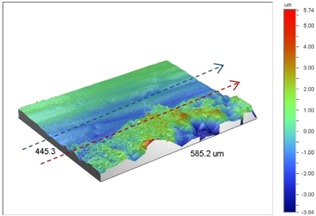	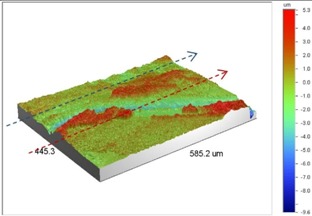
Line scan profiles	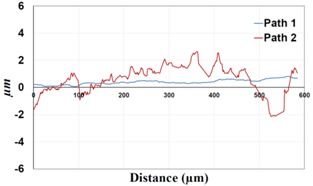	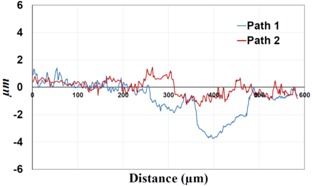

**Table 2:** 3D surface profile together with line scan profile for uncoated and coated aluminum plates clamped with 5 and 8 Nm that failed under maximum remote stress of *S_max_* = 80 MPa.

Profiles	Uncoated Al 7075-T6 Plates	
	*T* = 5 Nm	*T* = 8 Nm
3D surface profile	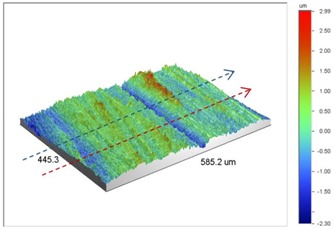	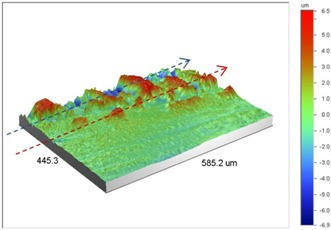
Line scan profiles	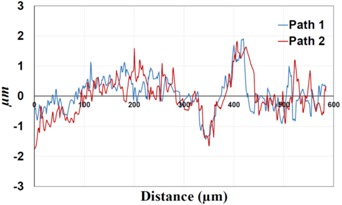	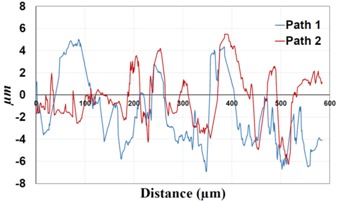
**Profiles**	**Coated Al 7075-T6 plates with Ni–P coatings**	
	***T* = 5 Nm**	***T* = 8 Nm**
3D surface profile	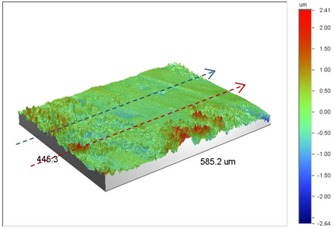	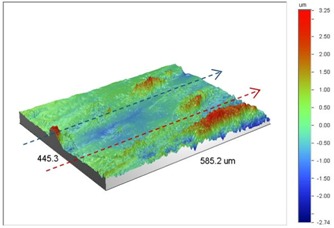
Line scan profiles